# Ludwik Maurycy Hirschfeld (1814–1876)

**DOI:** 10.1007/s00415-020-10102-3

**Published:** 2020-07-29

**Authors:** Ignacy Gonkowski, Slawomir Gonkowski, Krystyna Makowska

**Affiliations:** 1 Students Science Club of Pathophysiologists. Department of Physiology and Pathophysiology. School of Medicine, University of Warmia and Mazury, Olsztyn, Poland; 2 Department of Clinical Physiology. Faculty of Veterinary Medicine, University of Warmia and Mazury, Olsztyn, Poland; 3 Department of Clinical Diagnostics, Faculty of Veterinary Medicine, University of Warmia and Mazury, Olsztyn, Poland

**Keywords:** Hirschfeld, Neuroanatomy, Neurology, Anatomy

Ludwik Maurycy Hirschfeld (Fig. 1) was born in a poor Jewish family on April 3, 1814 in Nadarzyn near Warsaw [1, 2]. His father was a shechita – man who dealt with Jewish ritual slaughtering [1]. Ludwik often watched his father during his work, thanks to which he became interested in anatomy [1]. In 1825 the Hirschfeld family moved to Warsaw, where Ludwik studied at Talmudic school [1, 2]. Probably at that time he started to think about medicine as a profession and began self-education in biology and chemistry [1, 2].

**Fig. 1:**
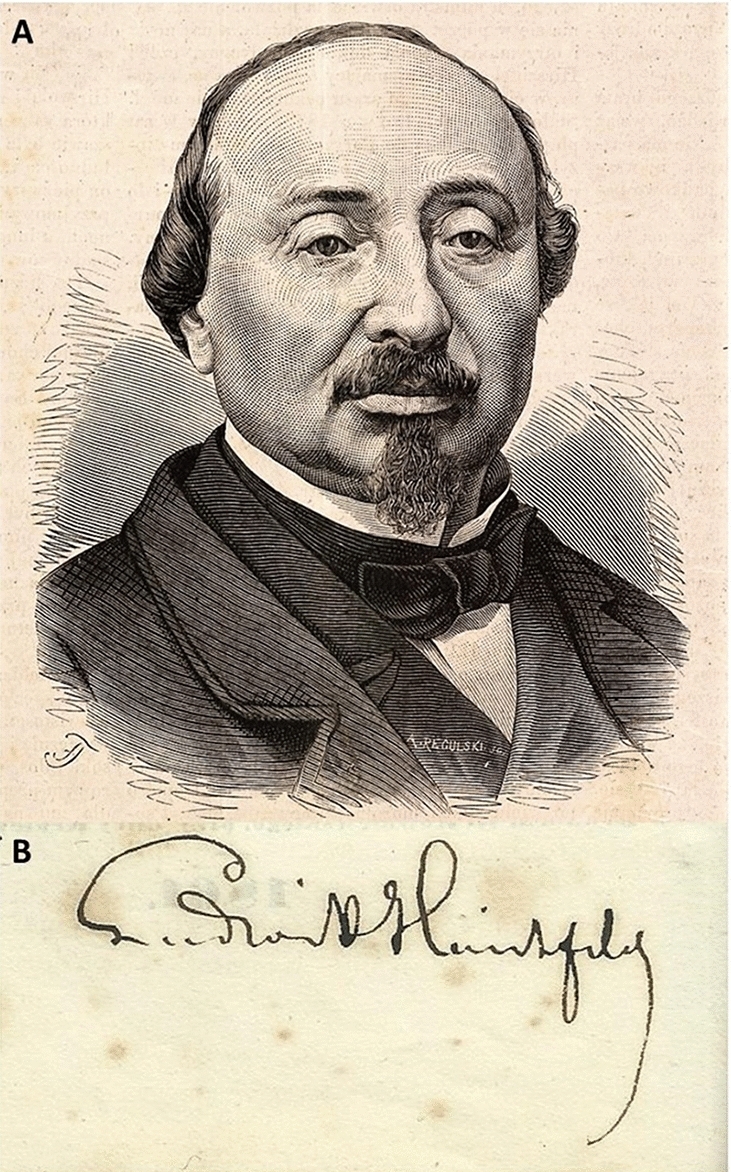
Ludwik Maurycy Hirschfeld—portrait from 1876 (public domain) (**a**) and his original autograph from private collection of S. Gonkowski (**b**)

In 1833 Hirschfeld, secretly from his family, went to Wrocław to start medical studies [1]. Because he had not graduated from any secondary school, Hirschfeld was not admitted to the university and came back to Warsaw [2]. However, he did not give up his dreams of medicine and in the same year he ran away from home again. This time he went to Berlin. Due to lack of money he walked all the way. From Berlin Hirschfeld continued his journey to Paris and after walking through the whole Germany he reached his destination in 1834 [2].

In Paris, he started to work as a dogsbody in the anatomical studio directed by Jean-Baptiste Marc Bourgery [1]. Initially Hirschfeld dealt with scraping and whitening bones [1, 2], but later he did more complicated preparations. In his spare time, Hirschfeld attended the lectures in anatomy and did anatomical preparations for his private purpose [2]. The legend says that during one of the lectures Hirschfeld presented his own preparation to Bourgery, who, impressed with the excellence of preparation, said to the audience: “on your knees, gentlemen!” [2]. After that Hirschfeld started work as a dissector [2]. Moreover, the Dean of Paris Medical Faculty, Mathieu Orfila, obtained permission from the Ministry of Education for Hirschfeld’s study at the Medical Faculty without secondary school graduation [1].

In this way, in 1848, at the age of 34, fourteen years after he arrived in Paris, Ludwik Maurycy Hirschfeld fulfilled his dream and took the degree of doctor of medicine on the basis of a dissertation entitled “Des injectionis capillaires” [2]. A year later, as a “private professor”, he started to hold lectures on anatomy [2]. In 1853, Hirschfeld together with the artist Jean Leveille published an atlas of the nervous system entitled “Neurology or description and iconography of the nervous system and human sense organs with their mode of preparation” [3]. The atlas comprised 92 modern and precise illustrations of the anatomical preparations made personally by Hirschfeld [2, 3] and won recognition from contemporary neurologists [2]. Hirschfeld received an award from the French Academy of Sciences, became an honorary member of many scientific societies, and in 1857 he was appointed a director of the clinic headed by famous internist Leon Rostan at Paris Hôtel-Dieu hospital [2]. In the clinic and in his private medical practice Hirschfeld primarily dealt with neurological diseases [1, 2, 4].

However, despite the honors, Hirschfeld missed his homeland and his parents at their advanced age [2]. Therefore, he willingly accepted the invitation of the newly established Caesarian-Royal Medical-Surgical Academy in Warsaw and on January 21, 1859 he was appointed a head of the Department of Anatomy at this academy [2]. In the same year, September 15, 1859, Hirschfeld began lecturing in anatomy [5] and soon he made himself known as a great teacher [2]. He was an expert in the smallest details and secrets of anatomy, but simultaneously he was gentle and sympathetic to his students [2]. Hirschfeld reformed Polish anatomical nomenclature, presented in the first modern Polish-language anatomical book, “Descriptive human anatomy”, published in 1861 [1, 2]. One volume of this book, “Neurology, i.e. a description of the human nervous system and sensory devices along with physiological notes”, entirely devoted to the nervous system, was the first modern Polish manual on neuroanatomy and neurophysiology [6]. Hirschfeld also dealt with scientific work and he spent every vacation in a chosen scientific centre in Europe [2]. Moreover, he led a medical practice. He enjoyed the recognition of patients who often called him “a general” [2].

The hard work and long years spent in the anatomical studio impaired Hirschfeld’s health [1]. In May 1875, due to poor health, he asked for dismissal from the position of university professor and permission to retire [1]. The Medical Committee concluded that Hirschfeld suffered from heart diseases, pulmonary edema and hemiparesis [2]. Ludwik Hirschfeld died on May 10, 1876, only one and a half months after his last lecture for students [1, 2].

Ludwik Maurycy Hirschfeld was primarily an excellent anatomist dealing first of all with the nervous system and was called by internist Leon Rostan “the head of all anatomists” [1]. His neuroanatomical atlas of 1853 was one of the first such accurate and modern descriptions of the anatomy of the nervous system [3]. Figures contained in this work were later used in atlases and manuals of the most prominent anatomists, including Johannes Sobbota, Ludwik Edinger, and August Rauber [2]. In turn Hirschfeld’s “Descriptive human anatomy” of 1861, written in Polish language, was a pioneering book which induced modern anatomical nomenclature into Polish medicine [6]. Hirschfeld was also the author of several articles about various aspects of human anatomy published in scientific journals [2, 7, 8] and the inventor of the “medullotom”, a tool for the better preparation of the medulla oblongata [9]. However, Hirschfeld was also a clinician. He was one of the first surgeons, who dealt with the diagnosis of brain tumours and abscesses within the central nervous system on the basis of neurological symptoms [2, 4, 10]. Moreover, he was the author of probably the first description of the set of symptoms connected with the foramen lacerum resulting from the pressure on the cranial nerves, later known as Avellis syndrome [2].

Ludwik Maurycy Hirschfeld enjoyed great recognition among his contemporaries. In 1876 the famous surgeon and professor of Warsaw University Polikarp Gisztowt wrote: “Ludwik Maurycy Hirschfeld was a man of strong will, steadfast and extraordinary diligence. The world granted him the title of scientist and in fact he was a scientist. His name will never stop living in the history of anatomy.” [1].

## References

[1] Girsztowt P (1876). Dr Ludwik Maurycy Hirschfeld [in Polish]. Tygodnik Ilustrowany.

[2] Bychowski A (1929) Ludwik Maurycy Hirschfeld—professor of anatomy (1814-1876) [in Polish]. Druk. Koop. Prac. Druk, Warsaw

[3] Hirschfeld L, Leveille JB (1853). Neurology or description and iconography of the nervous system and human sense organs with their mode of preparation [in French].

[4] Hirschfeld l (1859). Brain tumors diagnosed diagnosed during life [in Polish]. Rocznik Towarzystwa Paryskiego Lekarzy Polskich.

[5] Hirschfeld L (1859). The first lecture on descriptive anatomy [in Polish]. Tygodnik Lekarski.

[6] Hirschfeld L (1861). Neurology, i.e. a description of the human nervous system and sensory devices along with physiological notes [in Polish].

[7] Hirschfeld L (1860). About uterine nerves [in Polish]. Pamiętnik Towarzystwa Lekarskiego Warszawskiego.

[8] Hirschfeld L (1865). New view on anatomy of vertebral column curvatures [in Polish]. Gazeta Lekarska.

[9] Hirschfeld L (1865). Medullotom [in Polish]. Przegląd Lekarski.

[10] Hirschfeld L (1866). Abscess in posterior mediastinum adjacent to the arachnoid mater of spinal cord [in Polish]. Klinika.

